# Plasma Tau Levels in Cognitively Normal Middle-Aged and Older Adults

**DOI:** 10.3389/fnagi.2017.00051

**Published:** 2017-03-06

**Authors:** Ming-Jang Chiu, Ling-Yun Fan, Ta-Fu Chen, Ya-Fang Chen, Jen-Jei Chieh, Herng-Er Horng

**Affiliations:** ^1^Department of Neurology, National Taiwan University Hospital, College of Medicine, National Taiwan UniversityTaipei, Taiwan; ^2^Graduate Institute of Brain and Mind Sciences, College of Medicine, National Taiwan UniversityTaipei, Taiwan; ^3^Department of Psychology, National Taiwan UniversityTaipei, Taiwan; ^4^Graduate Institute of Biomedical Engineering and Bioinformatics, National Taiwan UniversityTaipei, Taiwan; ^5^Section of Neurology, Department of Psychosomatic Medicine, Taipei City HospitalTaipei, Taiwan; ^6^Department of Medical Imaging, National Taiwan University Hospital, College of Medicine, National Taiwan UniversityTaipei, Taiwan; ^7^Institute of Electro-Optical Science and Technology, National Taiwan Normal UniversityTaipei, Taiwan

**Keywords:** age, immunomagentic reduction assay, plasma tau, biomarker, normal cognition

## Abstract

Using an ultra-sensitive technique, an immunomagnetic reduction assay, the plasma tau level can be measured to a limit of quantification of pg/ml. In total 126 cognitively normal middle-aged and older adults (45–95 years old) were recruited. The plasma tau levels were significantly higher in the older group (aged 65–95 years) 18.14 ± 7.33 pg/ml than those in the middle-aged group (aged 45–64 years) 14.35 ± 6.49 pg/ml when controlled gender and *ApoE*ε4 carrier status (*F* = 3.102, *P* = 0.029). The *ApoE*ε4 carriers had higher plasma tau levels than the non-carriers when controlled age and gender (*F* = 6.149, *P* = 0.001). Men had higher plasma tau levels than their women counterparts when controlled *ApoE*ε4 carrier status and gender (*F* = 6.149, *P* = 0.001). The plasma tau levels were found to be positively associated with their ages (*r* = 0.359, *P* < 0.001). Regression analysis showed that age explained approximately 13% of the variance in the plasma tau levels, and explained more than 10% of the variance in the volumes of the hippocampus and white matter hypodensity (*R*^2^ change 0.123~0.167, all *P* < 0.001), and explained less than 10% of the variance in the volume of the amygdala, and central part of the corpus callosum (*R*^2^ change 0.085~0.097, all *P* = 0.001). However, the plasma tau levels do not further explain any residual variance in the volume of brain structures. In conclusion, the effect of age on the plasma tau levels should always be considered in clinical applications of this surrogate biomarker to middle-aged and elderly subjects.

## Introduction

Diagnosis of preclinical Alzheimer’s disease (AD) or other suspected non-Alzheimer pathologies depends on the evidence of genetic carrier status and pathophysiological changes in the brain, such as shown by fluid and imaging biomarkers. Neuronal injury or neurodegeneration-associated biomarkers provide clinicians to monitor progression of cognitive impairment, to predict prognosis, and to evaluate therapeutic outcomes of disease-modifying interventions in clinical trials (Buerger et al., 2009). Elevation of the tau level in cerebrospinal fluid (CSF) may suggest active axonal and neuronal destruction (Tapiola et al., 2009). Patients with AD were reported to have three-fold higher CSF tau levels (Hampel et al., 2008); some considered it is a potential biomarker of AD (Lewczuk et al., 2017); and even might predict a rapid progression from mild cognitive impairment (MCI) to AD-associated dementia (van Rossum et al., 2012). However, in addition to patients with AD, increased total tau levels in the CSF of patients with certain other neurological diseases, such as acute stroke, Creutzfeldt-Jakob disease and natalizumab-treated multiple sclerosis (Otto et al., 1997; Hesse et al., 2000; Kapaki et al., 2001; Llorens et al., 2016; Mellergård et al., 2017). Acute traumatic brain injury may also cause increased release of tau protein into the CSF (Zemlan et al., 1999).

The tau level in the CSF has been associated with hippocampal atrophy in some studies (Hampel et al., 2005; Desikan et al., 2011; de Souza et al., 2012). The tau level in the CSF also correlates to severity of brain atrophy in the abovementioned natalizumab-treated multiple sclerosis patients at the 3-year follow-up (Mellergård et al., 2017).

The plasma tau levels of patients with neurological diseases have not been well studied until recently. Recent studies have found that plasma tau levels were increased during the acute phase (Randall et al., 2013) and late phase (Mortberg et al., 2011) of hypoxic brain injury following cardiac arrest. A study on Olympic boxers, who suffered from repetitive non-knocked-out head concussions from boxing bouts, plasma tau levels were elevated within 1–6 days of a bout and returned to normal after resting at least for a period of 14 days (Neselius et al., 2013). Zetterberg et al. (2013) applied a single molecule array (Simoa^TM^) assay to quantitatively evaluate the total tau protein level in plasma and found that the plasma tau protein concentration of AD patients is higher than those of MCI patients and normal controls. Chiu et al. (2013) assayed the plasma tau protein level using an immunomagnetic reduction assay and showed that the levels of plasma tau protein are higher in patients with MCI due to AD and in patients with AD dementia than in normal controls.

However, no study has explored the effect of aging on the plasma tau protein level in normal population due to the even lower concentration of tau protein in the plasma of normal subjects. The aims of the present study were to elucidate the effect of age on the plasma tau levels of cognitively normal middle-aged and older adults and to explore possible correlations between brain structures and the plasma tau protein levels in these cognitively normal subjects.

## Materials and Methods

### Subjects

The volunteers who participated in this study were asked to complete a medical checklist regarding major systemic diseases, operations and hospitalizations. Volunteers who reported having uncontrolled medical conditions, including heart failure, a recent myocardial infarction (within the past 6 months), malignancy (within the past 2 years) or poorly controlled diabetes (HbA1C > 8.5), were excluded. Volunteers also received physical and neurological examinations and were scored on a short-form geriatric depression scale (GDS-S). Those who had a GDS-S score of greater than 9 were excluded. All of the participants provided written informed consent prior to participating in this investigation. The study was approved by the ethics committee and the institutional review board of the National Taiwan University Hospital (protocol 201301036-RIND). The study was carried out in accordance with the Helsinki Declaration of 1975.

### Cognitive and Functional Assessments

The assessments performed in this study included determining the clinical dementia rating (CDR) scores and the scores on the short-form geriatric depression scale (GDS-S); additionally, the Taiwanese mental status examination (TMSE), two subtests of the Wechsler memory scale (WMS)-III logical memory subtest and family picture subtest were performed (Hua et al., 2005). For the cognitive assessments, the participants had to be within normal limits (scores higher than 1.5 standard deviation) of age- and education-matched normal subjects. None of the participants met the diagnostic guidelines for all causes of dementia or MCI proposed by the NIA-AA workgroups in 2011 (Albert et al., 2011; McKhann et al., 2011).

### Structural MRI

High-resolution brain structural brain MRI scans were acquired using a 1.5 T MRI scanner (EXCITE, General Electric, Milwaukee, Wisconsin, USA). A whole-brain T1-weighted 3D spoiled gradient recovery (SPGR) sequence was used (TE = 9.3 ms, TR = 3.9 ms, TI = 600 ms, flip angle = 12°, matrix size = 192 × 192 and FOV = 25 cm), and 170 images of contiguous sagittal slices that were 1.3 mm in thickness were acquired. The analyzed datasets include the T1-weighted structural MRI scans obtained from all of the available subjects (*n* = 123). The MRI images were corrected for intensity inhomogeneity and were reoriented, after which they were registered against MNI 152 space using FSL (FMRIB Software Library) FIRST tools (Smith et al., 2004). Image analysis to determine the cortical thickness, gray matter volume and white matter hypodensity were performed using the FreeSurfer software package (version 5; Athinoula A. Martinos Center for Biomedical Imaging, Boston, MA, USA) on an AAL template.

### Plasma Tau Protein Assays

Participants were asked to provide a 10-ml non-fasting venous blood sample (K3 EDTA, lavender-top tube). The blood samples were centrifuged (2500× g for 15 min) within 1 h of collection, and the plasma was aliquoted into cryotubes and stored at −80°C for less than 3 months until it was thawed for measurement of the tau levels via an immunomagnetic reduction assay. The reagent (MF-TAU-0060, MagQu) used to determine the plasma tau protein levels consisted of dextran-coated Fe_3_O_4_ nanoparticles functionalized with antibodies directed against tau protein. In brief, the anti-tau antibody (Sigma, T9450) was derived from mouse monoclonal clone tau46 which recognizes bovine, rat, human and mouse tau. This monoclonal antibody also recognizes a phosphorylation-independent epitope in amino acids 404–441 (human) and all six isoforms of tau thus measuring total tau. The mean diameter of the nanoparticles was 53 nm. The antibody-functionalized nanoparticles are well dispersed in a phosphate-buffered saline solution of pH 7.2. A SQUID-based ac magnetosusceptometer (XacPro-S, MagQu) was used to analyze the samples. A 60-μl aliquot of plasma was mixed with 60 μl of reagent at room temperature for determination of the tau protein concentration. The underlying mechanism and the technological details of the immunomagnetic reduction assay are described in our previous reports (Chiu et al., 2012; Yang et al., 2013).

### Statistical Analyses

Independent *t*-test was used to examine between-group difference of parametric variables of demographic and clinical data. Pearson chi square was used to examine distribution of non-parametric data such as gender and ApoE**ε**4 carrier status. One-way ANCOVA (analysis of covariance) was used to compare plasma tau levels between different age groups (middle-aged vs. older subjects), genders and ApoE**ε**4 carrier status. Pearson correlation test was applied to identify brain structures as significant dependent variables of stepwise linear regression analysis. Stepwise linear regression analysis was used to evaluate the percentage of the variance explained by the independent variables.

## Results

We recruited 126 cognitively normal adults aged 45–95 years (mean: 66.7 ± 9.6) of whom 61.1% were women. The subjects included 56 middle-aged (45–64) and 70 older (65–95) adults (Table 1). There was a significant difference in the plasma tau levels between the middle-aged and older adults (*F* = 3.102, *P* = 0.029) when controlled for the effects of gender and ApoE**ε**4 carrier status. There was also a significant between-group difference in the plasma tau levels of ApoE**ε**4 carriers (17.29 ± 6.89 pg/ml) and non-carriers (16.27 ± 7.28 pg/ml, *F* = 6.149, *P* = 0.001) when controlled for the effects of age and gender. Gender also significantly affected the plasma tau levels (men 17.11 ± 7.16 pg/ml, women 16.05 ± 7.23 pg/ml, *F* = 6.149, *P* = 0.001) when controlled for the effects of age and ApoE**ε**4 carrier status.

**Table 1:** **Demographic and clinical information of the participants**.

Subjects (numbers)	All (126)	Middle-aged (56)	Older (70)
Years of age (range)	66.7 ± 9.6 (45–95)	58.1 ± 4.9 (45–64)	73.6 ± 6.3 (65–95)
Gender women %	61.6	70.9	54.3*
Years of education	13.2 ± 3.7	13.5 ± 3.5	13.0 ± 3.9*
TMSE score	28.5 ± 1.6	29.0 ± 1.3	28.2 ± 1.8
ApoEε4 (%)	18.3	14.3	21.4*
Plasma tau (pg/ml)	16.46 ± 7.2	14.35 ± 6.49	18.14 ± 7.33^†^

TMSE, Taiwan mental status examination; ApoEε4, prevalence of apolipoprotein Eε4; *No significant between-group difference in items listed; ^†^Significant group difference when controlled for gender and ApoEε4 carrier status by ANCOVA, F = 3.102, P = 0.029.

**Figure 1:**
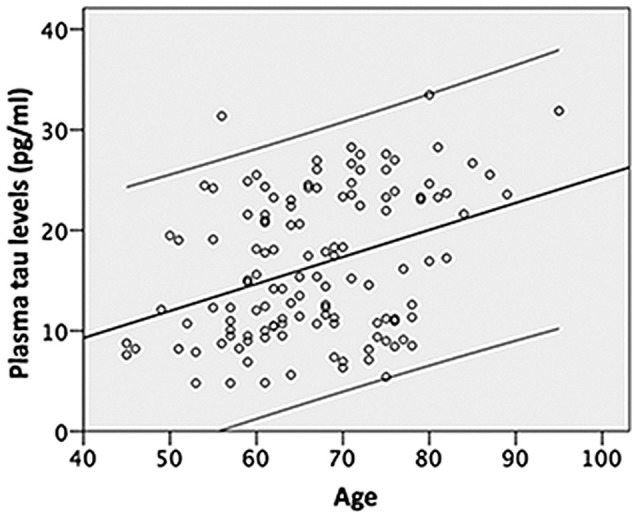
**Shows that age is positively associated with the plasma tau level and explains approximately 13% of the variance (*R*^2^ change 0.129, *F* = 18.329, *P* < 0.001) in the plasma tau levels; the upper and lower lines indicate the 95% confidence intervals**.

Correlation analyses showed that age was negatively correlated with the TMSE scores (*r* = −0.543, *P* < 0.001), whereas age was positively correlated with the plasma tau levels (Pearson *r* = 0.359, *P* < 0.001; Figure 1). We performed the Bonferroni adjustment to control the family-wise error rate derived from multiple comparisons of brain structures (*P* defined as 0.001). Image analyses showed that age was significantly negatively correlated with the volumes of the bilateral hippocampus (left *r* = −0.362 and right *r* = −0.408, both *P* < 0.001), bilateral amygdala (left *r* = −0.311 and right *r* = −0.294, both *P* = 0.001), and the volumes of the central part of the corpus callosum (*r* = −0.291, *P* = 0.001), whereas age was positively correlated with the volume of white matter hypodensity (*r* = 0.351, *P* < 0.001). A partial correlation analysis was performed to explore the relationship between plasma tau levels and measurements of brain structures by controlling age effect. The plasma tau levels were not significantly correlated with both the volume of the subcortial brain structures and thickness of various cortical regions when controlled age effect.

Further analyses using stepwise linear regression showed that age explained approximately 13% of the variance in the plasma tau levels (*R* square (*R*^2^) change 0.129, *F* = 18.329, *P* < 0.001) excluded the years of education, gender and ApoE4 carrier status (Figure 1). In addition, age explained more than 10% of the variance in the hippocampal volumes (*R*^2^ change 0.1321, *F* = 17.836 for the left and *R*^2^ change 0.167, *F* = 23.607 for the right, both *P* < 0.001) and white matter hypodensity (*R*^2^ change 0.123, *F* = 16.561, *P* < 0.001) excluded the years of education, gender, plasma tau levels and ApoE4 carrier status (Table 2). ApoE4 carrier status explained only additional 3% of the variance of the right hippocampal volume (*R*^2^ change 0.03, *F* = 4.390, *P* = 0.038; Table 2). Age explained less than 10% of the variance in the amygdala volume (*R*^2^ change 0.097, *F* = 12.622 for the left and *R*^2^ change 0.086, *F* = 11.131 for the right, both *P* = 0.001) excluded the years of education, gender, plasma tau levels and ApoE4 carrier status (Table 2).

**Table 2:** **Stepwise multiple linear regression analyses with measurements of brain structures as dependent variables and age, gender, education and tau as independent variables**.

Brain structure_Model_	*R*^2^ change	*F*	Significance
^R^Hippocampus_Vol Model I Age_	0.167	23.607	<0.001
^R^Hippocampus_Vol Model II *ApoE*ε4_	0.03	4.390	0.038
^L^Hippocampus_Vol Model I Age_	0.131	17.836	<0.001
^R^Amygdala_Vol Model I Age_	0.086	11.131	0.001
^L^Amygdala_Vol Model I Age_	0.097	12.622	0.001
CC central_Vol Model I Age_	0.085	10.949	0.001
WM hypodensity_Model I Age_	0.123	16.561	<0.001

*TMSE, Taiwanese mental status examination; ^L/R^, left/right; CC, corpus callosum; Vol, volume*.

## Discussion

Recently Mattsson et al. (2016) reported that higher plasma tau associated with AD dementia, higher CSF tau and lower CSF Aβ. Thus, plasma tau levels may at least in part reflect the CSF tau levels. A previous study found a correlation between age and the CSF tau level (Sjögren et al., 2001). The aging-related elevation of the plasma tau protein levels found in the cognitively normal middle-aged and older adults in this study might be a consequence of the normal aging process of the brain. The elevated plasma tau levels may imply the increasing production of tau protein in the brain with increasing age. This hypothesis is compatible with the age-related increment in tau related pathology, such as tangle formation and neuronal loss, even in non-demented aging individuals (Ball, 1977; Terry et al., 1987; Price and Morris, 1999). Plaques were absent in some of the brains of the non-demented elderly, and the earliest plaque formation in other brains was found to have occurred in the neocortex, in patches of diffuse plaques (Price and Morris, 1999). This type of aging-related structural change was also reflected in the findings of the present study, in which quantitative aspects of some brain areas, such as the volumes of the hippocampus, amygdala and the central part of the corpus callosum were negatively associated with the age, whereas the volume of white matter hypodensity was positively associated with the age. However, the regional specificity and age-related linearity of age-related brain volume changes remain controversial (Terribilli et al., 2011). Although tau protein levels might reflect a certain degree of neuronal loss and consequent regional brain atrophy, this phenomenon can be well explained in great part by the aging process because stepwise regression analysis showed that the brain structural values was mostly explained by the aging effect and no additional effect was explained by increasing or decreasing plasma tau protein levels. In an interesting contrast, plasma tau levels were found to be negatively correlated with the hippocampal and brain volumes of patients with MCI due to AD or mild AD (Chiu et al., 2014) but not with those of cognitively normal controls in this study. This result might imply that the plasma tau level is useful as a surrogate biomarker representing disease severity rather than only the effect of aging and that it can be used to monitor disease progression or beneficial responses to current disease-modifying therapies.

The ApoE**ε**4 carriers had higher plasma tau levels than did their non-carrier counterparts. ApoE**ε**4 carrier status significantly increases the risk of developing AD via both amyloid-related and non-amyloid-related mechanisms (Kanekiyo et al., 2014). ApoE**ε**4 promotes amyloid deposition in the brain, which may further result in the appearance of tau in the brain through the “Aβ-induced tau pathology” mechanism (Stancu et al., 2014). Gender also had an effect on the plasma tau levels that men had higher plasma tau levles than their women counterparts. A recent *in vitro* study showed that the male sex hormone testosterone was more efficient in lessening Aβ-induced mitochondria deficits, while the women sex hormone progesterone and estrogen were the most effective neurosteriods in AD related tauopathy (Grimm et al., 2016).

To the best of our knowledge, this is the first report demonstrating the tau levels in the blood of cognitively, neurologically and psychiatrically normal middle-aged and older individuals. Knowledge of the tau level in the blood is more useful than is knowledge of that in the CSF due to the much easier access to blood and the greater clinical convenience and safety of blood collection. One of the most important strength of the present study is that our ultra-sensitive IMR method (limit of detection lower than pg/ml) is able to demonstrate the age effect on tau levels in the blood. The plasma levels of tau measured in this study are higher (about twofold) than those in the previous studies using either conventional enzyme-linked immunosorbent assay (ELISA) or other ultra-sensitive technology such as the Simoa^TM^ digital ELISA (Zetterberg et al., 2013). The reasons may include that IMR is more resistant to the interference from other components in the blood such as albumin or bilirubin; the preparation of our sample is quite straightforward avoiding loss of target protein during multi-step processing; also our tau antibody recognizes all isoforms of tau therefore achieve higher measures (Chiu et al., 2013; Yang et al., 2013).

However, this study is not without limitations. First, because our subjects included middle-aged to elderly adults but did not include young adults, such as those with ages in the range of 20–44 years old, care must be taken in generalizing the results of age effect of the present report. Second, this was a cross-sectional study, and a longitudinal study of the same individuals over a long period, such as 5–10 years, might validate our findings of the age-related increase in the plasma tau level. Also, lack of longitudinal follow-up, there is a potential risk of including subjects with preclinical neurodegenerative disease such as AD or other tauopathy into the study population. The current study, therefore, warrants a long-term follow-up study. Third, we did not have data of the CSF tau levels to cross-validate our findings because Asian people, particularly older people, are rather reluctant to submit to a lumbar puncture. Also information of CSF and/or plasma Aβ42 or p-tau is not available in this study. However, this phenomenon indicates the importance of our report because the ultra-sensitive assay we employed allows quantifying the ultra-low tau levels in the blood and allows a safer and more comfortable assessment of the level of a neuronal-injury biomarker.

## Conclusion

The study demonstrated that age affects the level of plasma tau protein the same as the level of CSF tau protein found in a previous study (Sjögren et al., 2001). Thus, we must consider the age effect when using either the plasma or CSF tau level as a surrogate biomarker for axonal/neuronal damage or degeneration.

## Author Contributions

M-JC: idea formation, statistical analysis, manuscript drafting and finalizing. L-YF: imaging processing and image data analysis, statistical analysis. T-FC: subjects recruitment, data interpretation. Y-FC: imaging acquisition, image quality control, image processing. J-JC: IMR measurement, IMR data preparation. H-EH: idea formation, supervision of IMR measurement. All authors listed, have made substantial, direct and intellectual contribution to the work, and approved it for publication.

## Conflict of Interest Statement

The authors declare that the research was conducted in the absence of any commercial or financial relationships that could be construed as a potential conflict of interest.
